# Factors associated with the rural and remote practice of medical workforce in Maluku Islands of Indonesia: a cross-sectional study

**DOI:** 10.1186/s12960-021-00667-z

**Published:** 2021-10-09

**Authors:** Farah Noya, Sandra Carr, Sandra Thompson, Rhonda Clifford, Denese Playford

**Affiliations:** 1 Division of Health Professions Education, School of Allied Health, University of Western Australia, Perth, Australia; 2 Medical Education Unit, Faculty of Medicine, Pattimura University, Ambon, Indonesia; 3 Western Australian Centre for Rural Health, The University of Western Australia, Perth, Australia; 4 School of Allied Health, University of Western Australia, Perth, Australia; 5 The Rural Clinical School of WA, School of Medicine, The University of Western Australia, Perth, Australia; 6 Health Professions Education Building, The University of Western Australia, Crawley Avenue (off Mounts Bay Road-next to CAR PARK 25), Nedlands, WA 6009 Australia

**Keywords:** Archipelagic context, Developing country, Medical workforce, Rural background, Regional medical school, Recruitment and retention of rural doctors, Rural and remote practice, Rural intention, Rural preference

## Abstract

**Background:**

Many factors contribute to engagement in rural and remote (RR) medical practice, but little is known about the factors associated with rural and remote medical practice in such remote locations as the Maluku Province of Indonesia. This study describes factors associated with actual RR practice, preferred RR practice, and intention to remain practice in Maluku Province.

**Methods:**

An online survey of work-related experience and intentions for future rural work was administered to 410 doctors working in the Maluku province of Indonesia. Participant characteristics were described using descriptive statistics, associations between the independent variables with the location of the workforce, intention to remain practice in Maluku, preference for future RR practice in Maluku were analysed using Chi-square tests and logistic regression.

**Results:**

A total of 324 responses (79% response rate) were recorded, comprising 70% females and 30% Pattimura University graduates of doctors employed in Maluku. Doctors working in RR areas were more likely to be a GP (OR 3.49, CI 1.03–11.8), have a monthly salary of more than IDR 6 million (OR 11.5, CI 4.24–31.1), and have no additional practice (OR 2.78, CI 1.34–5.78). Doctors intended to stay practice in Maluku were more likely to be born in Maluku (OR 7.77, CI 3.42–17.7) and have graduated from Pattimura University (OR 3.06, CI 1.09–8.54), and less likely to be a temporary employee (OR 0.24, CI 0.10–0.57). Doctors who prefer future RR practice in Maluku were more likely to experience rural living (OR 2.05 CI 1.05–3.99), have a positive indication of the impact of community exposure during medical schools on their current practice (OR 2.08, CI 1.06–4.09), currently practising in RR Maluku (OR 8.23, CI 3.27–20.8); and less likely to have bigger take-home pay (OR 0.30, CI 0.13–0.70).

**Conclusion:**

This study indicates that special attention should be given to recruiting doctors with a rural background and ongoing support through attractive opportunities to build a sustainable RR workforce. Since a regional medical school helps supply doctors to the RR areas in its region, a sustained collaboration between medical schools and local government implementing relevant strategies are needed to widen participation and improve the recruitment and retention of RR doctors.

**Supplementary Information:**

The online version contains supplementary material available at 10.1186/s12960-021-00667-z.

## Background

A shortage of health personnel and workforce maldistribution means unequal access to healthcare for people living in rural and remote (RR) communities, a persistent and significant problem globally [1–3]. Indonesia, a middle-income country in the Asian region, struggles with community access to healthcare and insufficient health workforce, especially in its RR areas [4–6]. Indonesia has 29% of its districts categorised as underdeveloped and has almost three-fourth of its areas classified as rural [7]. Having ratio of doctors per population 1:2294 nationally, its health data shows maternal mortality rate 177/100,000 live births [8], higher than the World Health Organisation (WHO) target in the context of the Sustainable Development Goals (SDGs) [9], and neonatal mortality rate 15 deaths/1000 live births [8], higher than the SDGs target 12/1000 live births [10]. Despite these poor outcomes, the national government allocates just 5% of its budget for health [11]. Service coverage index of Universal Health Coverage (UHC) through Indonesian National Health Insurance was 60% in 2020, with higher UHC achieved mostly in Java Island and some western parts of Indonesia [12].

Maluku, a province in the eastern Indonesian archipelago, includes some of the most remote, isolated and poorly served islands [8, 13, 14]. According to the Ministry of Health of the Republic of Indonesia, the characteristic of the working area of a health service facility is classified into urban, rural, remote and very remote areas. Maluku health service facilities were determined remote, very remote, and unattractive [15], underdeveloped (73% of the province districts) [13], and in the outermost islands (17% of national numbers) [14] include challenging conditions. They are: located in areas that are difficult to reach or prone to disasters; small islands, island clusters, or coastal regions; have poor routine public transportation access (once per week); long travel times to the district/city capital (more than 6 h round trip); travel may be hindered by the climate or weather; experience difficulties in fulfilling essential commodities; and may have unstable security conditions [15].

In addition to the remote and isolated nature of the province being a disincentive for medical personnel choosing to live and work there [4, 16, 17], the region has less infrastructure, facilities and amenities, difficulties with communication, perceived lower quality of children’s education and lower employment income [4, 16, 17]. In Indonesia, the national health data analysis shows that the number of doctors in an area was positively related to population numbers, population density, number of hospitals and community health centres [17]. The ratio of doctors per population is 1:7269 in Maluku. In recent years, the central government has implemented various policies such as compulsory work placements and financial and career incentives to attract and bond doctors and health professionals to remote and isolated areas of Indonesia through Temporary Assignment and Nusantara Sehat schemes [4, 16, 18]. Even though Maluku has been one province, where doctors may extend their stay after compulsory work placements with government incentives [19], the small numbers of doctors in RR areas of Maluku remains a significant issue [8].

Subsequently, Maluku has national data showing maternal mortality rates (> 177/100,000 live births), infant mortality rates (> 22 deaths/1000 live births) and infectious diseases rates that are many times higher than in countries with acceptable access to care [8, 20]. In this province, the service coverage index of UHC was only 55% [12].

Maldistribution of rural health personnel, particularly doctors, occurs globally [2, 21–23]. Recruitment and retention of doctors in rural areas is influenced by many factors, including personal characteristics such as rural background and educational elements including rural exposure during medical training. A scoping review of 61 papers published between 2010 and 2020 [24] concluded that rural background and rural training are decisive factors in recruiting and retaining doctors in RR areas [25–49].

In Indonesia, before establishing Pattimura University Medical School in Ambon, Maluku, doctors who worked in Maluku predominantly graduated from medical schools in Java Island (Jakarta, Surabaya, Yogyakarta, and Bandung) and cities outside Java Island, such as Medan, Makassar and Manado. In 2008, an undergraduate medical school was established in Pattimura University to educate medical students to work locally in Maluku. The government partly funded the first five cohorts of 50 students to encourage graduates to serve the province, with more than 20% of students having a rural background [50]. The vision was to improve the number of doctors (an additional 50) in Maluku by 2015.

Understanding factors associated with the medical workforce taking up RR practice in Indonesia, specifically Maluku, is limited and not adequately addressed in Indonesian and international research. Hence, this study aimed to investigate factors related to doctors’ preferences to work in the RR areas of Maluku Province. Specifically, this research answers three research questions:

1. What are the characteristics of doctors who work in Maluku?
2. Which demographic variables are associated with current RR location of practice, intention to remain to practise in Maluku, and RR practice preference in Maluku?

Besides being relevant as an evaluation of the current program in Pattimura University, findings from this study can inform policy and practice for other archipelago regions and other low- and middle-income countries, especially within the Asian region with similar societal and regional geographical characteristics.

## Methods

### Study population

The study population comprised qualified doctors who were currently working in Maluku Province.

#### Inclusion and exclusion

Doctors employed in Maluku (hospitals and health clinics, administrators and academics), of any age, gender and discipline/specialty area were included. Those temporarily unemployed or working in another province were excluded.

#### Sampling

Based on the data recap provided by the Health Human Resources division of Maluku Province Health Office, 496 doctors worked in Maluku. However, the given list provided 440 names. After ethical and governance approvals from The University of Western Australia, Pattimura University and the Maluku Province government, all doctors were invited to participate in the study. Their names and contact details were provided by the provincial and regents health offices and the Pattimura University Medical School alumni database.

#### Recruitment

After excluding 30 doctors who were on other assignments outside Maluku during the survey period, 410 doctors were identified as working in Maluku, 133 (32%) males and 277 (68%) females. All were invited to participate in the study via a text message to the mobile number provided by the health offices and medical school. This message contained information about the study with a link to the online survey. Consent to participate was embedded in the online survey.

### Instruments

The 51-item survey developed by the researchers was informed by the literature (Additional file 1: Survey questionnaire) and included multiple-choice, dichotomous, multi-response and Likert type questions. The sections included:

1. Demographic details, including the rural background (rural born, Maluku born, rural living experience and the length of rural living experience).
2. Medical training history, including rural exposure.
3. Employment status and history, including practice location defined using the Indonesian Centre Bureau of Statistic classification for urban and rural areas in Maluku [7], with the site of practice converted to the category (rural = 1 or urban = 0).
4. Outcome variables, i.e., current practice is in RR areas, intended to remain practice in Maluku, and preferred future RR practice in Maluku.

### Data collection

Online-based delivery was the most feasible option given the doctors’ geographical distribution. The survey was open for 2 months to mitigate limited internet access in the Maluku area.

### Data analysis

All analyses were undertaken based on valid cases using IBM SPSS statistics version 26. Descriptive statistics were used to determine participants’ characteristics. A Shapiro–Wilks test was used to confirm the normality of the data distribution. Chi-square tests were performed after a test of independence between outcome variables and independent variables (Tables 2, 3 and 4) to determine the relationships between both. The outcomes of interest were defined as:

1. The current workplace is in RR Maluku areas (0 = No, 1 = Yes)
2. The intention to remain working in Maluku (0 = No, 1 = Yes).
3. The preferred future location of practice in Maluku is RR areas (0 = No, 1 = Yes).

A test of multicollinearity was performed using *r* value for Spearman Correlation. When two variables had significant and positive strong relationship (*r* value of more than 0.7), selection for further analysis was based on the significance of the variables. Binary logistic regression was based on variables with a significance level < 0.20 to estimate odds ratios associated with factors determine the target outcomes. A confidence interval of 95% and alpha significance level of 0.05 were used. A further collinearity diagnostic was perform using variance inflation factor (VIF) in linear regression with all variables in three outcomes of interest showed VIF around 1.

## Results

### Characteristic of respondents

**Fig. 1:**
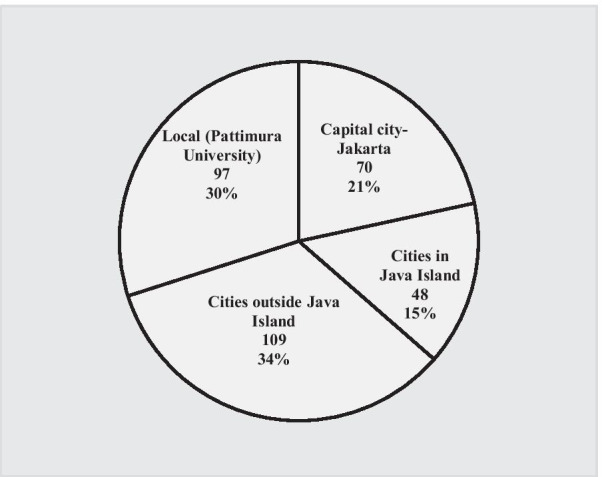
Location of participants’ medical schools

There were 324 doctors who recorded responses, so 79% of eligible participants commenced the survey, with 241 (59%) completed surveys. Non-completion was reported by some of the respondents mainly due to problems with internet stability. Respondents were more likely to be young (mean 33.4 years), female, married, and Maluku born (Table 1). Of the females, 61% were working in a RR practice location. Of all respondents born in Maluku, less than a third were born in RR areas. More than half the respondents had never lived in rural areas before commencing medical school. Most doctors graduated from medical schools in cities outside Java (Fig. 1), and almost all respondents experienced some rural exposure during their medical program (Table 1).

**Table 1:** Characteristics of respondent doctors working in Maluku Province

	*N*^a^ (%)
Medical training history	
Medical school origin	
All else	227 (70)
Pattimura University	97 (30)
Community exposure during medical training	
No	9 (3)
Yes	315 (97)
Indicated impact of community exposure^b^	
Negative	34 (12)
Passive	155 (54)
Positive	98 (34)
Rural exposure	
No	33 (11)
Yes	254 (89)
Demographic variables	
Gender	
Male	76 (29)
Female	183 (71)
Age (23–66 years, mean 33.4 STDEV 8.4)	
Less than mean	157 (65)
More than mean	84 (35)
Marital status	
Married	133 (51)
Unmarried	126 (49)
Have child/ren under care	
Yes	107 (41)
No	152 (59)
Rural born	
No	189 (76)
Yes	60 (24)
Province of birth is Maluku	
No	89 (36)
Yes	159 (64)
Rural living experience	
No	147 (59)
Yes	102 (41)
Length of rural living experience	
Less than 10 years	179 (72)
Minimum of 10 years	69 (28)
Employment variables	
Employment status	
Permanent	117 (44)
Temporary	149 (56)
Specialisation in medicine	
General practitioner (GP)	249 (86)
Medical specialist	39 (14)
Length of work since graduation^c^	
More than 5 years	117 (41)
Up to 5 years	171 (59)
Length of work in current practice^c^	
More than 5 years	86 (30)
Up to 5 years	202 (70)
Monthly salary (Mean IDR 5,917,765, Stdev 4,575,245)	
IDR 6 million and less	178 (67)
(IDR 1 million–IDR 29 million)	
Greater than IDR 6 million	88 (33)
Additional practice	
No	182 (68)
Yes	84 (32)
Take-home pay^d^ (Mean IDR 11,928,497, Stdev 13,390,630)	
IDR 12 million and less	182 (69)
(IDR 1.5 million–IDR 150 million)	
Greater than IDR 12 million	83 (31)
Outcome variables	
Current practice is in rural and remote Maluku areas	
No	118 (41)
Yes	169 (59)
Preferred future location of practice^e^	
Outside Maluku	58 (23)
Urban Maluku	128 (52)
Rural Maluku	62 (25)

^a^Not all participants answered every question so the numbers do not add to 324

^b^Adjusted to dichotomous with a median of 8 (0 = less positive, 1 = more positive)

^c^Highly associated with younger age, excluded from the regression model

^d^Take-home pay here includes medical service fees in Indonesian case-based group (INA-CBGs) and or capitation within Indonesian National Health Insurance, also practice and other service fees

^e^Adjusted for outcome 2: intended to remain in Maluku for future practice (0 = No, 1 = Yes); adjusted for outcome 3: those who prefer rural and remote practice in Maluku (0 = No, 1 = Yes)

**Fig. 2:**
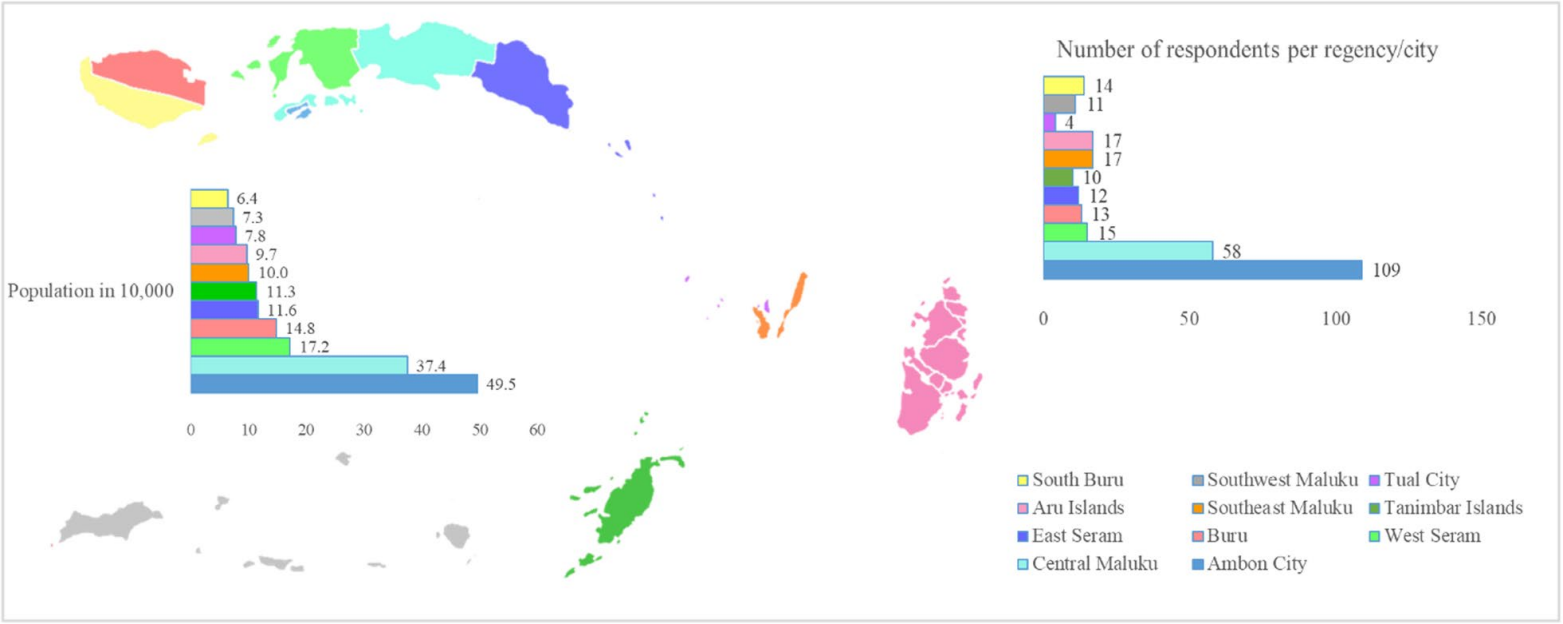
Map of Maluku with population and respondents number per regency/city

Most respondents had worked less than 5 years since graduation and under 5 years in their current post, had only temporary contracts, and did not undertake additional practice besides their main job (Table 1). Of the 11 regencies in Maluku, respondents predominantly worked in Ambon (39%), the capital city of Maluku, and the nearest regency from the capital, Central Maluku regency (21%) (Fig. 2).

**Fig. 3:**
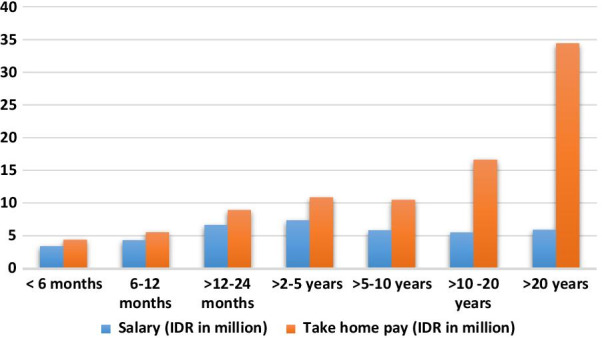
Average monthly salary and take-home pay over the duration of work

Figure 3 shows that there was no significant difference in salary between durations of work and that take-home pay was higher for those who had worked for more than 10 years. Cross tabulation of the monthly wage with a specialty in medicine showed that more general practitioners (GP) received a salary of more than 6 million IDR (90% vs 84% who received ≤ 6 million IDR). Meanwhile, for take-home pay, more GPs received up to 12 million IDR (97% vs 57% who received more than 12 million IDR). Take-home pay includes medical service fees in Indonesian case-based groups (INA-CBGs) and or capitation* within Indonesian National Health Insurance as well as practice and other service fees. The analysis of duration of work with monthly take-home pay and location of current work and additional practice showed that of doctors who had worked for more than 10 years, 50% had an additional practice, and 78% were located in more developed urban areas, Ambon city (59%) and Central Maluku Regency (15%). In addition, those doctors with take-home pay greater than IDR 12 million were mainly of older age (OR 6.41, CI 95% 3.46–11.9, *p* = 0.000), permanent employees (OR 4.45, CI 95% 2.50–7.92, *p* = 0.000) and specialists (OR 27.5, CI 95% 10.1–74.8, *p* = 0.000) who need specialist facilities so are permitted to have up to an additional two practices [51].

**Fig. 4:**
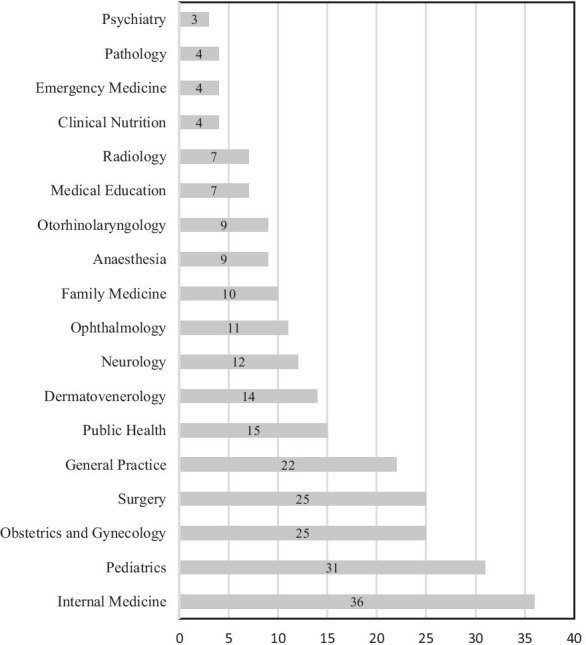
Participant’s future practice type preference

More than half the respondents stated their preferred future location for work was in the urban areas of Maluku, with only a quarter nominating to stay in RR posts in Maluku (Table 1). Four essential specialty areas in Indonesian Medicine (Internal Medicine, Paediatrics, Obstetrics and Gynaecology, Surgery) were the commonly chosen future disciplines, with Internal Medicine the most favoured (36; 14.5%; Fig. 4).

### Rural and remote practice in Maluku Province (Table 2)

Univariate analysis showed strong associations between the current rural practice location with younger age (≤ 33 years), Pattimura graduates, general practitioners, monthly salary more than IDR 6 million, and having no additional practice. These associations were not significant in the multivariate model. There was no association between rural practice location with rural born, Maluku born, and rural exposure. Multivariate logistic regression showed that doctors currently practice in a RR location were more likely to; be a GP (OR 3.49, CI 1.03–11.8), have a monthly salary of more than IDR 6 million (OR 11.5, CI 4.24–31.1), and have no additional practice (OR 2.78, CI 1.34–5.78).

**Table 2:** Variables associated with the current rural and remote practice location

	*N* valid cases	Location of practice		Univariate		Multivariate	
		Rural (%)	Urban (%)	p*	OR (CI 95%)	p*	OR (CI 95%)
Demography							
Age (less than mean 33.35)	240	73 (mean 32)	53 (mean 36)	**0.002**	**2.36 (1.37**–**4.08)**		
Gender (female)	258	70	73	0.616	0.87 (0.50–1.51)		
Marriage status (single)	258	54	39	**0.022**	**1.81 (1.09**–**3.02)**		
Have child/ren under care (No)	258	65	49	**0.010**	**1.95 (1.17**–**3.02)**		
Rural background							
Rural born	248	26	21	0.317	1.37 (0.74–2.54)		
Province of birth is Maluku	247	63	66	0.556	0.85 (0.50–1.46)		
Have rural living experience	248	44	36	0.232	1.38 (0.81–2.35)		
More than 10 years of rural living experience	100	52	50	0.477	1.24 (0.69–2.22)		
Medical training and community exposure							
Pattimura medical school origin	287	36	23	**0.017**	**1.90 (1.12**–**3.24)**		
Multistages of learning using community exposure	279	53	43	0.112	0.47 (0.09–2.36)		
Positive impact of community exposure to medical practice	245	43	34	0.187	1.42 (0.84–2.41)		
Multi foci of community exposure	279	73	64	0.100	1.54 (0.92–2.56)		
Experienced rural exposure during medical training	279	90	86	0.391	1.37 (0.66–2.85)		
Employment factors							
Specialty in medicine (GP)	287	90	81	**0.037**	**2.05 (1.04**–**4.06)**	**0.044**	**3.49 (1.03**–**11.8)**
Temporary employment status	265	61	50	0.079	1.56 (0.95–2.57)		
Monthly salary more than IDR 6 million	265	46	13	**0.000**	**5.97 (3.09**–**11.5)**	**0.000**	**11.5 (4.24**–**31.1)**
Having no additional practice	265	23	45	**0.000**	**2.73 (1.60**–**4.65)**	**0.006**	**2.78 (1.34**–**5.78)**
Monthly take home pay more than IDR 12 million	265	35	24	**0.046**	**1.76 (1.01**–**3.09)**	0.055	3.04 (0.98–9.44)

The bold emphasises the significance of the variables and their ORs

^*^The Chi-square statistic is significant at the 0.05 level

### Intention to remain practice in Maluku Province (Table 3)

Univariate analysis showed three factors positively associated with intention to remain practice in Maluku Province; Maluku born (OR 11.4, CI 5.65–23.1), rural exposure during medical school (OR 2.46, CI 1.10–5.48), and Pattimura University graduates (OR 4.25 CI 1.83–9.88). With OR less than 1, being younger doctors (OR 0.41, CI 0.20–0.85), having temporary employment status (OR 0.33, CI 95% 1.19–3.98), and having no additional practice (OR 0.27, CI 95% 0.12–0.60) were negatively associated with intention to stay practice in Maluku. In other words, those who intend to stay were more likely doctors who were older, in permanent employment, and having additional practice. Multivariate analysis revealed that doctors intended to stay practice in Maluku were more likely to be born in Maluku (OR 7.77, CI 3.42–17.7), and have graduated from Pattimura University (OR 3.06, CI 1.09–8.54), and less likely to have temporary employment status (OR 0.24, CI 0.10–0.57).

**Table 3:** Variables associated with intention to stay practice in Maluku Province

	*N* valid cases	Intention to remain practice in Maluku Province		Univariate		Multivariate	
		Yes (%)	No (%)	p*	OR (CI 95%)	p*	OR (CI 95%)
Demography							
Gender (female)	248	70	69	0.881	1.05 (0.56–2.00)		
Age (less than mean 33.35)	231	62	80	**0.014**	**0.41 (0.20**–**0.85)**		
Marriage status (single)	248	48	59	0.153	0.65 (0.36–1.18)		
Have children under care (No)	248	57	74	**0.018**	**0.46 (0.24**–**0.88)**		
Rural background							
Rural born	248	26	21	0.526	1.26 (0.62–2.58)		
Province of birth is Maluku	247	77	22	**0.000**	**11.4 (5.65**–**23.1)**	**0.000**	**7.77 (3.42**–**17.7)**
Have rural living experience	248	42	38	0.621	1.17 (0.64–2.13)		
More than 10 years of rural living experience	248	19	22	0.562	0.81 (0.40–1.66)		
Medical training and community exposure							
Graduated from Pattimura Medical School	248	37	12	**0.000**	**4.25 (1.83**–**9.88)**	**0.033**	**3.06 (1.09**–**8.54)**
Multistage learning using community exposure	241	51	44	0.378	1.31 (0.72–2.38)		
Indicated positive impact of community exposure to medical practice	241	39	25	0.053	1.93 (0.99–3.78)		
Multifocal community exposure	241	73	61	0.100	1.69 (0.90–3.15)		
Experienced rural exposure during medical training	241	90	79	**0.024**	**2.46 (1.10**–**5.48)**		
Employment factors							
General practitioners	248	83	91	0.124	0.47 (0.17–1.26)		
Temporary employment status	248	51	76	**0.001**	**0.33 (0.17**–**0.65)**	**0.001**	**0.24 (0,10**–**0.57)**
Monthly salary more than IDR 6 million	248	31	33	0.807	0.92 (0.49–1.73)		
Having no additional practice	248	63	86	**0.001**	**0.27 (0.12**–**0.60)**	0.066	0.38 (0.17–1.07)
Monthly take home pay more than IDR 12 million	248	31	24	0.348	1.38 (0.70–2.72)		
Currently work in rural/remote Maluku	247	62	66	0.670	0.88 (0.47–1.62)		

The bold emphasises the significance of the variables and their ORs

*The Chi-square statistic is significant at the 0.05 level

### Rural and remote preference of practice in Maluku Province (Table 4)

There were positive associations between younger age, rural born, Pattimura graduates experienced multistage, multifocal community exposure during medical school, indicated a positive impact of community learning on current practice, being a GP, practising RR currently, with the preferred location of practice being rural and remote Maluku. Having take-home pay more than 12 million IDR was negatively associated with rural preference among those intended to stay in Maluku. Controlling for confounding variables, doctors who prefer rural practice in Maluku were more likely to have rural living experience (OR 2.05, CI 1.05–3.99), have a positive indication of the impact of community exposure during their medical schools on their current practice (OR 2.08, CI 1.06–4.09), have current practice in RR Maluku (OR 8.23, CI 3.27–20.8), and less likely to have monthly take-home pay more than IDR 12 million (OR 0.30, CI 0.13–0.70).

**Table 4:** Variables associated with rural and remote preference among doctors who intended to remain practise in Maluku

	*N* valid cases	Prefer RR Maluku for future practice		Univariate		Multivariate	
		Yes (%)	No (%)	*p*	OR (CI 95%)	*p*	OR (CI 95%)
Demography status							
Age (less than mean 33.35)	231	77	62	**0.039**	**2.02 (1.03**–**3.95)**		
Gender (female)	248	76	68	0.231	1.49 (0.77–2.88)		
Marriage status (single)	248	57	48	0.271	1.38 (0.78–2.47)		
Have child/ren under care (No)	248	68	59	0.202	1.48 (0.81–2.72)		
Rural background							
Rural born	248	34	20	**0.031**	**2.00 (1.06**–**3.77)**		
Province of birth is Maluku	247	74	61	0.053	1.87 (0.99–3.56)		
Have rural living experience	248	41	36	**0.009**	**2.16 (1.20**–**3.86)**	**0.036**	**2.05 (1.05**–**3.99)**
More than 10 years of rural living experience	248	23	19	0.519	1.26 (0.63–2.53)		
Medical training and community exposure							
Graduated from Pattimura University	248	47	26	**0.002**	**2.53 (1.39**–**4.59)**		
Multistages of learning using community exposure	241	63	44	**0.010**	**2.18 (1.20**–**3.98)**		
Indicated positive impact of community exposure to medical practice	241	48	31	**0.015**	**2.09 (1.15**–**3.79)**	**0.033**	**2.08 (1.06**–**4.09)**
Multifocal community exposure	241	82	66	**0.024**	**2.26 (1.10**–**4.67)**		
Experienced rural exposure during medical training	241	90	87	0.507	1.38 (0.53–3.55)		
Employment factors							
General practitioners	248	94	82	**0.031**	**3.13 (1,06**–**9.22)**		
Currently work in rural/remote Maluku	248	90	54	**0.000**	**7.72 (3.17**–**18.8)**	**0.000**	**8.23 (3.27**–**20.8)**
Temporary employment status	247	65	54	0.160	1.53 (0.84–2.77)		
Monthly salary more than IDR 6 million	248	44	32	0.090	1.66 (0.92–3.00)		
Having no additional practice	248	76	66	0.135	1.64 (0.85–3.17)		
Monthly take home pay more than IDR 12 million	248	21	36	**0.034**	**0.48 (0.24**–**0.93)**	**0.005**	**0.30 (0.13**–**0.70)**

The bold emphasises the significance of the variables and their ORs

^*^The Chi-square statistic is significant at the 0.05 level

## Discussion

This survey of doctors living and practising in the Maluku Province of Indonesia offers valuable insights into factors significant to recruiting and retaining a sustainable rural workforce.

This study identified that doctors currently working in Maluku were predominantly female, of young age, married, did not have a rural background, graduated from a university outside Maluku, and experienced rural exposure. They mainly were working rurally, GPs, held temporary contracts, at early stage career, received salary up to IDR 6 million and take-home pay up to IDR 12 million, and have no additional practice.

Doctors currently working in rural and remote Maluku were more likely to be general practitioners with salaries more than IDR 6 million and no additional practice. Doctors intending to remain in Maluku Province were more likely Maluku born, Pattimura graduates, and permanent workers. Doctors who preferred RR Maluku for their future practice were more likely to have current practice in RR Maluku and rural living experiences and less likely to have take-home pay over IDR 12 million.

This study identified more doctors practising rural were female (70%). This finding is notably different from other international [27, 36, 52] and current Indonesian [53] studies describing characteristics of RR doctors, where male doctors tend to be the majority. However, another Indonesian study with a similar archipelagic context found that females were dominant (59%) in the rural workforce [54]. While not showing association in this sample, another study in Australia has reported that more women are applying to Rural Clinical Schools [40] and proportionately more are going on to rural work [32], thus beginning to redress rural workforce shortages in female practitioners.

The majority of doctors practising in Maluku graduated from medical schools in regional or provincial capitals, including almost a third from Pattimura University, Maluku. Given that most of our respondents were Maluku-born, this finding confirms that regional students come back to their regions [55–57], and implies that significant efforts should be put into developing regional medical schools to improve doctors’ distribution to the regions [55–57].

Interestingly, in this study, rural exposure during medical training, one of the most widely reported factors in other studies, was not associated with RR practice location and preference for RR practice location in Maluku. This was also found in a study from Canada [58]. However, the study was from 1999 when medical schools were in the early stages of advancing rural experiences for their students. A current study with early-career Indonesian doctors showed that doctors practising in remote locations were likely to have a clerkship in a remote district [53]. And a 10-year longitudinal cohort study from Australia revealed that rural exposure during medical training related to rural work [59].

While not significantly associated with rural practice location and rural preference in this study, we found that most doctors experienced rural exposure during their medical training, more than what was found in the national study [53]. This is similar to the Australian requirement that all students experience rural work during their medical training [59]. Moreover, we found that rural exposure was associated with more than twofold rates of intention to remain practice in Maluku. More directed positive strategies are needed.

Another important factor reported elsewhere as a determinant of RR practice uptake is rural background [25–41, 53], but it was not associated with rural practice location in this study. However, rural born and rural living experiences were associated with the preference of future rural practice location in Maluku, with rural living experience independently associated (twofold rates) with the preference. We also found that Maluku born, although not meaning rural born, was associated significantly with the intention to stay practice in Maluku Province. All of these factors should inform the development of pro-rural work policies for this archipelago.

These positive policies are not only the domain of Western countries, as we confirm that a geographical maldistribution exists even in this developing province [60]. However, since our findings suggest that being rural born were associated with the intention to remain practice in Maluku, more focus and attention should be given to the recruitment of students and doctors with a rural background. Although this finding is not novel, this study confirms the pattern among the limited number of studies from low- and middle-income countries.

In addition, evidence suggests that widening access to medical courses enhanced care to underserved communities [61–65]. A more comprehensive approach is needed to widen the participation and aspirations for medicine of under-represented socio-economic and educationally disadvantaged groups. This approach could include regent government early education programs and support, including scholarships aimed at these under-represented groups.

Regarding future practice type preference, doctors in Maluku preferred to work in specialist practices, namely, Internal Medicine, Paediatrics, Obstetrics and Gynaecology, and Surgery. Specialist practice, especially within the four major specialties, is an opportunity to earn more income and the four specialty areas align with international preferences among doctors and medical students [66–68]. We found that doctors currently practising RR were more likely to be a GP. Although there are currently few specialists in Maluku and there is a great need for more specialists, priority should be given to primary care, rural practice, rural generalist, and family medicine for these areas of practice are associated with improved recruitment and retention of RR medical human resources [27, 29, 30, 33, 34, 38, 69, 70].

We found that more doctors in Maluku Province received salaries up to IDR 6 million and take-home pay up to IDR 12 million. Moreover, monthly salary and take-home pay were relatively low regardless of the length of work (Fig. 3. IDR 5 million, equal to USD 350/GBP 250 and IDR 34.5 million, equivalent to USD 2400 or GBP 1750, respectively). Salary more than IDR 6 million associated with more than 11-fold rates of current RR practice. This confirmed the importance of Indonesian government support through financial incentives included in the salary for doctors who practice rurally within Temporary Assignment and Nusantara Sehat schemes [4, 16, 18]. However, given the temporary status of many doctors, a higher salary appears unlikely to guarantee the sustainability of the RR medical workforce.

Meanwhile, preference for future RR practice in Maluku Province was associated with take-home pay less than IDR 12 million. Take-home pays more than IDR 12 million were related to the duration of practice of more than 10 years, additional practice and urban location. In addition, doctors who received a take-home pay of more than IDR 12 million were more likely of older age, permanent employees, and specialist medical doctors. Take-home pay includes medical service fees in Indonesian case-based groups (INA-CBGs) and or capitation within Indonesian National Health Insurance, also practice and other service fees. Those with permanent employment in health care centres or who have additional practice in primary clinics or private practices can benefit from capitation. Still, specialists get more payments from service fees in up to three hospitals and private practices [71]. As more primary clinics, private practices and hospitals were likely established in Maluku urban areas, and the UHC within Indonesian National Health Insurance in this province is still low [12], these benefits were less likely for doctors in RR settings. This means that RR practice in Maluku provides no promising rewards, highlighting the call from others internationally that meaningful reward for rural work is needed [72].

In addition, it is evident from this study that younger doctors (less than 33 years) were more likely to work rurally and intended to stay rurally, perhaps reflecting this generations’ ethical stances around the world [73, 74]. They were more likely to take up and prefer rural practice; however, they received smaller take-home pay and mostly held temporary contracts. The rural work experience requirement and recommendation from the rural government for scholarship in specialist training from the Ministry of Health [75] means these rural posts are likely to temporarily attract younger doctors to rural service in Maluku. However, retaining doctors in rural and remote Maluku Province requires more than financial incentives. Evidence from elsewhere shows that educational [69, 76–81], multidimensional [82–86], and professional development strategies [87, 88] improves retention of doctors in RR areas. Multidimensional strategies [82–86] include the provision of infrastructure, facilities, and transportation—basic needs for health service improvement—the most challenging factors in the RR areas for medical doctors, which are less concerned in the remote and isolated islands and areas of Maluku Province [13–15]. Consequently, a collaboration between medical schools and local government is required to ensure relevant strategies are implemented to improve the recruitment and retention of doctors in RR areas.

This study shows that Pattimura University’s graduate was significantly associated with all three outcome variables and independently associated with the intention to remain practice in Maluku. From this result, it can be said that Pattimura University has successfully produced doctors willing to serve in the RR areas of Maluku Province. This promising evidence supports the achievement of Pattimura University Medical School philosophy, akin to the vision of the Philippines medical school [89] and the vision for rural clinical schools in Australia [32, 40, 90].

Considerable evidence shows that a medical school intentionally established in a workforce shortage region pays much greater attention to the region's health status and concern. This is the case in both the developed [28, 91, 92] and developing world [56, 89, 93]. The medical school in The Philippines, Zamboanga [89], has a similarly rural, archipelago, and developing country context as Indonesia, so its findings are likely to be immediately relevant. This medical school showed that effective and sustainable medical education is possible in poor rural areas [89, 94]. Compared to James Cook University which strongly favours applicants with rural backgrounds and requires a commitment to work rurally after graduation [28, 91, 92], Pattimura University only stresses the philosophical value to practise rurally and offers rural exposure during medical training without any requirement for a rural background or commitment to work rurally.

From the successful experience of other universities [23, 32, 34, 56, 89, 93, 95], and based on this study’s findings, Pattimura University could expand even further into the region’s rural areas. Stressing the university’s values by increasing the proportion of students with a rural background and ensuring rural exposure is offered at different year levels for a range of disciplines during the medical course will likely increase the number of graduates serving the RR areas of Maluku Province.

These data inform an argument for dedicated government support of recruiting people from areas of medical workforce shortage into medical school and support for students and graduate doctors through training and attractive opportunities to sustain their practices. These can subsequently retain the doctors in areas of workforce shortage.

The findings of this current study align with the WHO Guidelines on health workforce development, attraction, recruitment and retention in rural and remote areas [96] that recommends the combining or bundling up of strategies. These strategies include admitting students from a rural background, bringing education to the rural and remote areas in the province, implementing a comprehensive rural and remote curriculum in medicine and appropriate incentives for doctors working in rural and remote areas of Maluku. For greatest success, these strategies should be in collaboration between Pattimura Medical School, local authorities, community and civil society.

### Study limitation

Pertaining to the sampling frame, we noted a difference in the number of doctors working in Maluku compare to the list provided from provincial and regents health offices and the medical school. There was no integrated database listing all doctors working in this province. The use of the Pattimura University alumni database to identify additional medical graduates augments the denominator for sampling but may potentially bias the participant sample.

Using a cross-sectional study, it is not possible to draw inferences of causality and outcomes of individual preferences and inclination to remain in rural practice, which can easily change. A longitudinal study is needed to track whether the participants are still in rural practice 5 to 10 years from now. Furthermore, due to the relatively large number of missing data despite the high response rate, this study may not have had the power to detect less strong associations between rural born and rural exposure in medical training and subsequent practice location.

This study only includes the graduates who currently work in Maluku province. Hence, there is a possibility of early-career bias to remain practising closer to where they trained. We realised that a further study is needed to track all Pattimura graduates workplace distribution across provinces in Indonesia.

The definition of rurality used in this study was the Indonesian national classification which may differ from other countries and make comparison challenging. Further studies on the RR workforce will benefit from applying the Degree of Urbanization, a United Nations (UN) recommendation on delineating cities, urban and rural areas for international statistical comparisons [97].

## Conclusion

This study indicates that to build a sustainable RR workforce in Maluku Province, special attention should be given to recruitment and retention of doctors with a rural background, and ongoing support through attractive opportunities to sustain their practices; and that a regional medical school helps supply doctors to the RR areas in its region. Sustained collaboration between medical schools and local government implementing relevant strategies are needed to widen participation and improve the recruitment and retention of rural and remote doctors.

## Supplementary Information

**Additional file 1.** Survey questionnaire.

## Data Availability

The data sets used and/or analysed during the current study are available from the corresponding author on reasonable request.

## Abbreviations

RR: Rural and remote
OR: Odds ratio
