# Supplementary material for: Factors associated with the rural and remote practice of medical workforce in Maluku Islands of Indonesia: a cross-sectional study
Source: Hum Resour Health. 2021 Oct 9;19:126. doi: 10.1186/s12960-021-00667-z (PMC8502290; doi:10.1186/s12960-021-00667-z)
Supplement: Supplementary file 1 — Additional file 1. Survey questionnaire. [file 12960_2021_667_MOESM1_ESM.pdf]

Professor Sandra Carr
Head,
Division of Health Professions Education
Faculty of Health and Medical Sciences
The University of Western Australia
35 Stirling Highway, Crawley WA 6009
Tel: 64886892
Email: sandra.carr@uwa.edu.au

Project title: The development, recruitment, and retention of the rural and remote medical workforce in the Maluku Province of Indonesia: A mixed-methods study.

Study 3. Determinant factors to the rural and remote medical workforce in the Maluku Islands: A cross-sectional study

Name of Researchers:

1. Prof Sandra Carr (Coordinating Supervisor & Chief Investigator)
2. Prof Sandra Thompson (Co-Supervisor)
3. Prof Rhonda Clifford (Co-Supervisor)
4. Assoc. Prof Denese Playford (Co-Supervisor)
5. Farah Christina Noya (Researcher and PhD Candidate)

Invitation

You are invited to participate in a project that seeks to describe the determinants of doctors' preferences to work in the rural and remote Maluku Province.
You are asked to take part in this project because you are a medical graduate that currently work in Maluku Province.

Aim of the Study:
This study aims at describing the determinants of doctors' preferences to work in the rural and remote Maluku Province.

What does participation involve?

THE UNIVERSITY OF WESTERN AUSTRALIA

If you agree to participate in the study, you will be asked to fill in an online-based questionnaire that link is sent with this email. The questionnaire will include questions related to:

1. Demographic details including rural background
2. Medical training history including rural exposure
3. Employment status and history
4. Workplace situation
5. Intention to work/remain in the rural and remote workforce for the next five years
6. Facilitations and barriers for rural and remote preference
7. Intrinsic motivational aspects

You may be invited to participate in a follow-up focus group discussion (FGD) or a semi-structured to further explore your thoughts around working in rural and remote Maluku Province

Voluntary Participation and Withdrawal from the Study

Participation in this study is voluntary. You can opt to withdraw from the study at early stage, without reason and consequence.

Your privacy

Your participation in this study and any information you provide will be treated in a confidential manner. The data obtained will be coded and de-identified before being stored in a password protected computer for a minimum of seven years. Video recordings will be deleted after transcription has been completed.

Findings from this study may be published or shared at professional gatherings, however at no time will any information be presented in such a way that will reveal your identity. Your anonymity will be protected.

Possible Benefits

There will be a draw prize of AU$100 each for four (4) respondents at the completion of the questionnaire sent to you. You may choose to enter the draw by providing your identity (email or phone number at the end of the questionnaire). Winners of the four prizes will be notified approximately 2 weeks after the survey closes. Please be aware that your contact details will not be aligned with your survey responses at any time. Your survey responses will remain anonymous.

THE UNIVERSITY OF WESTERN AUSTRALIA

Furthermore, It is hoped that the information gained through this study will inform the future support and development needs of the medical workforce in Maluku rural and remote areas.

Possible Risks and Risk Management Plan

There are no foreseeable risks associated with your participation in this research. Only de-identified data will be seen by the research team and will not be forwarded on to any other agency or used for any other purpose.

In the event you become distressed as a result of your participation you can opt to withdraw from this survey.

Contacts

If you would like to participate or discuss any aspect of this study please feel free to contact Farah on +61 420708210 or +62 811478210. Alternatively, you may contact Professor Sandra Carr on +61 6488 6892 or via email at sandra.carr@uwa.edu.au.

Sincerely,

Chief Investigator
Professor Sandra Carr
Phone: +61 8 6488 6892
Email: sandra.carr@uwa.edu.au

Researcher – PhD Candidate
Farah Christina Noya
Phone: +61 420708210 | +62 811478210
Email: farah.noya@research.uwa.edu.au

Approval to conduct this research has been provided by the University of Western Australia with reference number RA/4/20/5065, in accordance with its ethics review and approval procedures. Any person considering participation in this research project, or agreeing to participate, may raise any questions or issues with the researchers at any time. In addition, any person not satisfied with the response of researchers may raise ethics issues or concerns and may make any complaints about this research project by contacting the Human Ethics office at UWA on (08) 6488 4703 or by emailing to humanethics@uwa.edu.au. All research participants are entitled to retain a copy of any Participant Information Form and/or Participant Consent Form relating to this research project.

Consent

I have read the information provided, and any questions I have asked have been answered to my satisfaction. I agree to participate in this research project, realizing that I may withdraw at any time without reason and prejudice.
I understand that all identifiable information that I provide is treated as confidential and will not be released by the investigator in any form that may identify me unless I have consented to this. The only exception to this principle of confidentiality is if this information is required by law to be released.

I have been advised as to what data is being collected, the purpose for collecting data, and what will be done with the data upon completion of the research.

I agree that research data gathered for the study may be published or presented at professional gatherings, provided my name of other identifying information is not used.

I agree to participate in the survey.

1=Yes

2=No

I am interested in going into the draw for one of four prizes to the value of AU$100

1=Yes

2=No

I give consent to use my detail (email address or mobile number) separately for the prize draw

1=Yes

2=No

THE UNIVERSITY OF WESTERN AUSTRALIA

Start of Block: Medical Training History

1. What is the name of Medical School you graduated from?

____

2. In what year did you graduate from the Medical School?

____

3. Did you experience exposure with a local community (i.e., placement in a local community for any period as a part of studies) during your medical training?

1=Yes

2=No

If Yes, please proceed to Q number 4

If No, please proceed to Q number 9

4. When did the exposure take place?

1=Early pre-clinical years (year 1-2)

2=Late pre-clinical years (year 3-4)

3=The end of clinical years

4=Multiple stages during the clinical years

5=Other ____

5. Besides the exposure during the medical training, have you been exposed to these communities or come from/live in these communities?

1=Yes

2=No

6. For how long have you been exposed to these communities or come from/live in these communities?

1=Less than six months

2=6-12 months

3=>12months-2 years

4=>2-5 years

5=>5-10 years

6=>10-20 years

7=>20 years

7. Are the exposures positively affecting your current practice?

1=Yes

2=No

3= Not applicable

8. *In relation to question #7, PLEASE indicate how positive or how negative the impact to your current practice the exposure was with 1 being very negative and 10 being very positive.*

1	*2*	*3*	*4*	*5*	*6*	*7*	*8*	*9*	*10*

9. What was the focus of the attachment(s) (You can choose more than one option)

1= Community engagement

2= Clinical skills

3= Faculty community service

4= Other ____

10. What departments/units/blocks of learning have the community attachments? (You can choose more than one option)

1= Community/Public Health

2= Internal medicine

3= Pediatrics

4= Obstetrics and Gynecology

5= Surgery

6= Other= ___________________________________

How would you describe the place of your most community placements during medical training? Please tick the appropriate sections.

11. Location			12. Core Business		
Metropolitan/Urban (1)	Rural (2)	Remote/Island (3)	Hospital (1)	Public/community health centre (2)	Public/community health centre with inpatient facilities (3)

Start of Block: Employment Status and History

13. How long have you been working since you graduated from medical school?

1=Less than six months

2= 6-12 months

3= >12months-2 years

4= >2-5 years

5= >5-10 years

6= >10-20 years

7= >20 years

14. How long have you been working in your current workplace?

1=Less than six months

2=6-12 months

3=>12months-2 years

4=>2-5 years

5=>5-10 years

6=>10-20 years

7=>20 years

15. What is the name of your current practice and its location?

____

16. What is the discipline/specialty area of your practice?

1= Internal Medicine
2= Surgery
3= Obstetric and Gynaecology
4= Paediatrics
5= Emergency Medicine

6= Anaesthesia
7= Ear Nose and Throat
8= Ophthalmology
9= Neurology
10= Psychiatry
11= Pathology
12= Public Health
13= General Practice
14= Family Medicine

How would you describe your current place of employment?

17. Location			18. Core Business		
Metropolitan/Urban (1)	Rural (2)	Remote/Island (3)	Hospital (1)	Public/community health centre (2)	Public/community health centre with inpatient facilities (3)

19. What type of employment are you assigned in?
1=Permanent
2= Temporary with duration of more than 1 year
3= Temporary with duration of less than 1 year
4= Internship

20. What is your average daily working hours in the last year?

___

21.What are your average weekly working hours?

___

22. What is your monthly salary in IDR (Indonesian Rupiah)?

____

23. Do you undertake private practice?

    1=yes

    2= no

If you answer yes, please proceed to Q number 24

If not, please proceed to Q number 25

24. How many hours of private practice do you do per month?

____

25.What is your monthly take home pay (please include your salary, payment for private practice in the area and the estimated cost of other incentives if available) in IDR (Indonesian Rupiah)?

____

Start of Block: Facilitation and Barriers for remaining in Rural and Remote Practice

Please think about your last six months experience in your current workplace and tell us how much you agree with each of the following statements:

		Never	Sometimes	Often	Always	N/A
26	I get professional support and guidance from seniors and colleagues	◦	◦	◦	◦	◦
27	My workplace provides training and access to up to date clinical learning	◦	◦	◦	◦	◦
28	My workplace encourages my professional development	◦	◦	◦	◦	◦
		Strongly disagree	Disagree	Agree	Strongly agree	N/A
29	I am able to plan my career structure based on the employer management plan	◦	◦	◦	◦	◦
30	My employer gives me an opportunity to pursue medical specialty/academic qualification	◦	◦	◦	◦	◦
31	A scholarship is available for my medical specialty training/academic qualification	◦	◦	◦	◦	◦
32	My workload is realistic	◦	◦	◦	◦	◦
33	My employer defines clearly the scope of my practice and range of skills required	◦	◦	◦	◦	◦
34	My employer explains legal issues related to the required scope of	◦	◦	◦	◦	◦

	practice and indemnity					
35	My employer has appropriate incentives/pay according to my work commitment/based on the duration of service	◦	◦	◦	◦	◦
36	My workplace encourages social attachment	◦	◦	◦	◦	◦
37	My workplace is aware of my cultural skills and community engagement	◦	◦	◦	◦	◦
38	The health infrastructure of my workplace is adequate and meets contemporary standards	◦	◦	◦	◦	◦
39	My family's needs, such as schooling and amenities, are available in the local community	◦	◦	◦	◦	◦

40. Are there any other facilitators or barriers you would like to tell us about?

____

THE UNIVERSITY OF WESTERN AUSTRALIA

Start of Block: Motivational Aspects for Rural and Remote Practice

Read the following statements and tell us which items motivate you to work in the rural and remote areas. Mark 1- Yes if the factor is a motivator for you or 2-No if the factor is not a specific motivator for you

	I am motivated to work in rural and remote practice because:	1=Yes	2=No
41	I wish to contribute to the public good	◦	◦
42	I am interested in the challenge and satisfaction of working in a rural area	◦	◦
43	I like being useful for the rural community	◦	◦
44	I am inspired by a specific role model	◦	◦
45	I feel committed because of my rural background	◦	◦
46	I can see opportunities for career	◦	◦
47	I get adequate salary and incentives	◦	◦
48	I am stimulated by the diverse work	◦	◦
49	I am enjoying the lifestyle	◦	◦
50	I enjoy the benefits for my family	◦	◦
51	I have a rural doctor in my own family	◦	◦

Please tell us the **three** most important factors from the list below that help improve your job satisfaction in rural practice. Please insert the number of your choice to number 52, 53 and 54

1=Provision of subsidized housing
2=Supportive Management
3=Adequate facilities and equipment
4=Community appreciation of my contributions
5=Higher salary/incentive
6=Provision of training to support my skill development
7=Provision of scholarships
8=Making a difference in the community

52. ___________________________________
53. ___________________________________
54. ___________________________________

Start of Block: Personal Information

55. What is your gender?

1=Male

2=Female

56. What is your year of birth? _____ (YYYY)

57. Are you married?

1=Yes

2=No

If yes, please proceed to Q number 58

If no, please proceed to Q number 59

58. Is your spouse able to live with you in the location of your current practice?

1=Yes

2=No

59. Do you have a child/children?

1=Yes

2=No

If Yes, please proceed to Q number 60

If No, please proceed to Q number 62

60. How many children do you have?

____

THE UNIVERSITY OF WESTERN AUSTRALIA

61. How old are your children?

Child #1 ___________________

Child #2 ___________________

Child #3___________________

Child #4___________________

Child #5___________________

Child # ___________________

Please add to the list as necessary regarding the number of your children

62. How many of your children are in each stage below?

1=Preschooler ______________________
2=School-aged ______________________
3=University level ___________________
4=Working adult ____________________
5= Not working adult _________________

63. Have you ever relocated for your job because of your child's/children's schooling needs?

1=Yes

2=No

If yes, please proceed to Q number 64
If not, please proceed to Q number 65

64. When did you relocate?

1=as soon as my child/children started school
2= when my child/children started secondary school
3=when my child/children went to university

Start of Block: Rural background

65. Were you born in a remote/very remote/island area?

1=Yes

2=No

66. What is the province of your birth?

____

67. What is the Village area of your birth?

____

68. Didyou ever live in a rural/remote/island area prior to commencing medical school?

1=Yes

2=No

If Yes please proceed to Q number 69

If Not please proceed to Q number 72

69. How long did you live in a rural/remote/island area prior to commencing medical school?

____

70. In what stage of your life did you live in a rural/remote/island area prior to commencing medical school?

1= age 0-5 years old

2= Primary school

3= Junior high school

4= Senior high school

5= Other (or several stages, please specify) _____________________________

71. For what reason did you live in in a rural/remote/island area prior to commencing medical school?

1= it is my birthplace

2= it is my hometown

3= my parents got their assignment to the place

4= other reason

____

THE UNIVERSITY OF WESTERN AUSTRALIA

Start of Block: Intention to work/remain in the rural and remote workplace

72. I would prefer the location of practice for the next five years in

1= Another country

2= Urban areas of Indonesia outside Maluku

3= Rural areas of Indonesia outside Maluku

4= Urban areas in Maluku Province

5= Rural in Maluku Province

73. For my future practice type, I would prefer:

1= Internal Medicine

2= Surgery

3= Obstetrics and Gynaecology

4= Paediatrics

5= Emergency Medicine

6= Anaesthesia

7= Ear Nose and Throat

8= Ophthalmology

9= Neurology

10= Psychiatrists

11=Pathology

12= Public Health

13= General Practice

14= Family Medicine

Start of Block: Follow Up Invitation

The next phase of the study will focus on explaining and exploring the findings of the quantitative data. As per the information shared above, this phase will involve you participating in a Focus Group Discussion (FGD) or Interview.

If you consent to participate in the follow-up FGD/interview, please provide your details below to enable the researcher to contact you. Please note this information will not be used for any other purposes.

74. Name ____

75. Email Address ____

76. Phone Number (including country code)

____

You have reached the end of the questionnaire.
Thank you for participating in this survey.

If you consent to participate in the prize draw, please provide your details below to enable the researcher to contact you. Please note this information will not be used for any other purposes. Please be aware that your contact details will not be aligned with your survey responses at any time. Your survey responses will remain anonymous.

77. Name ____

78. Email Address ____

79. Phone Number (including country code)

____
